# New Families of Frequency-Hopping Sequence Sets with a Low-Hit-Zone

**DOI:** 10.3390/e26110948

**Published:** 2024-11-05

**Authors:** Limengnan Zhou, Hanzhou Wu

**Affiliations:** 1 School of Electronic and Information Engineering, University of Electronic Science and Technology of China, Zhongshan Institute, Zhongshan 528400, China; lmnzhou@zsc.edu.cn; 2 School of Information and Communication Engineering, University of Electronic Science and Technology of China, Chengdu 611731, China; 3 School of Communication and Information Engineering, Shanghai University, Shanghai 200444, China

**Keywords:** spread spectrum communication, frequency-hopping multiple-access, frequency-hopping sequence, low-hit-zone

## Abstract

As a means of spread spectrum communication, frequency-hopping technology has good performance in anti-jamming, multiple-access, security, covert communications, and so on. In order to meet the needs of different frequency-hopping multiple-access (FHMA) communication scenarios, the research on frequency-hopping sequence (FHS) sets with a low-hit-zone (LHZ) is now becoming more and more crucial. In this paper, a general construction to obtain new families of LHZ-FHS sets is achieved via interleaving technique. Subsequently, based on two different shift sequences, two classes of LHZ-FHS sets with new flexible parameters not covered in the related literature are presented. The requirements for our new LHZ-FHS sets to obtain optimality or near-optimality with respect to the Peng–Fan–Lee bound are also introduced. Furthermore, as long as the base FHS set is fixed, the performances of new LHZ-FHS sets can be analyzed, such that the parameters of all appropriate shift sequences to obtain desired LHZ-FHS sets are also fixed.

## 1. Introduction

With advantages such as anti-fading, anti-jamming, multiple-access and secure properties, frequency-hopping multiple-access (FHMA) systems have found wide applications in both military communications and civil communications [1,2]. Despite the physical goals of various FHMA systems, such as military radio communication systems, ultra wide bandwidth (UWB) radio systems, and echolocation communication systems, being considerably different, the demands imposed on the frequency-hopping signals are almost same. The function of frequency-hopping sequence (FHS) in such systems is to decide which frequency we should choose to transmit the information in each time slot. The carrier frequencies of two communication parties hop according to the agreed FHS set. It is hard for a third party who does not know the frequency-hopping rules in advance to discover or track the communication so as to achieve covert communication.

Usually, in FHMA systems, mutual interference (MI) occurs if more than one user transmits information on the same frequency simultaneously, and that may cause an increase in the possibility of destroying the communication process. Clearly, it is desirable to maintain the MI between transmitters at as low a level as possible. On the other hand, the Hamming correlation properties of the employed FHS sets are closely related to the degree of the MI, and so can be important evaluation criteria for the performances of FHMA systems [2,3]. Therefore, it is of great importance to design FHS sets with good Hamming correlation properties.

Unlike synchronous FHMA systems, in the quasi-synchronous (QS) ones, some relative time delay between different users within a zone around the origin can be tolerated. Meanwhile, for a low-hit-zone (LHZ) FHS set, its Hamming cross-correlations and autocorrelations are relatively small, so long as the time delay between different sequences does not exceed a limited zone known as LHZ [4]. Therefore, LHZ-FHS sets are well suitable for QS-FHMA systems. Furthermore, the research on both the theoretical lower bounds and the desired constructions of conventional FHS sets is comparatively rich [3,5,6,7,8,9,10,11]. Thus, in some sense, it is urgent to design more LHZ-FHS sets with both the desired Hamming correlation properties and new flexible parameters.

For an LHZ-FHS set, its maximum periodic Hamming correlation (PHC) within the LHZ, together with the LHZ, the sequence length, the sequence set size, and the frequency slot size, are limited by some mathematical formulas known as theoretical bounds. The first research into the theoretical bounds on LHZ-FHS sets can be dated back to the work of Peng et al. [4], in which the well-known Peng–Fan–Lee bound was derived. Thereafter, there have been a few designs of LHZ-FHS sets achieving or asymptotically achieving the Peng–Fan–Lee bound [12,13,14,15,16,17,18,19,20,21,22,23,24]. In this paper, via the interleaving technique, we also present two classes of LHZ-FHS sets with new flexible parameters not covered in the relevant literature, and which have optimality or near-optimality with regard to the Peng–Fan–Lee bound. For comparison, the parameters of our newly constructed LHZ-FHS sets and those of some known results are listed in Table 1.

So far, by the interleaving technique, some constructions of desired FHS sets have been reported by many researchers and scholars. In 2009, Chung et al. obtained some classes of optimal conventional FHS sets [25]. In 2012, Niu et al. obtained some classes of optimal LHZ-FHS sets [13]. In 2013, Liu et al. obtained a class of LHZ-FHS sets having optimal partial Hamming correlation properties [16]. Recently, the authors of the present paper also obtained a class of optimal or near-optimal LHZ-FHS sets [15]. Compared with the constructions in [13] or [15], those in this paper have different parameter constrains and shift sequences or different base FHS sets.

The rest of this paper is organized as follows. In Section 2, some related definitions and lower bounds on FHS sets are introduced. In Section 3, a new general construction to design LHZ-FHS sets through interleaving technique is presented, based on which two new classes of optimal or near-optimal LHZ-FHS sets are also proposed in Section 4. Finally, some concluding remarks are given in Section 5.

## 2. Preliminaries

Let $F=\{f_{0},f_{1},\cdots ,f_{\rho -1}\}$ be a frequency slot set with ρ available frequencies, $G=\{g^{0},g^{1},\cdots ,g^{\eta -1}\}$ be an FHS set with η frequency-hopping sequences of length ι over *F*, and each element of every sequence in *G* is in *F*. For two frequency-hopping sequences $g^{i}=[g_{0}^{i},g_{1}^{i},\cdots$, $g_{\iota -1}^{i}],g^{j}=\left[g_{0}^{j},g_{1}^{j},\cdots ,g_{\iota -1}^{j}\right]\in G,0\leq i,j<\eta$, their PHC function $H_{g^{i},g^{j}}\left(\tau \right)$ at time delay τ is defined as follows:

$$\begin{array}{ccc} H_{g^{i},g^{j}}\left(\tau \right)\; & = & \;\sum_{x=0}^{\iota -1}h\left[g_{x}^{i},g_{x+\tau }^{j}\right],\;0\leq \tau <\iota , \end{array}$$

where $h[g_{x}^{i},g_{x+\tau }^{j}]=1$ if $g_{x}^{i}=g_{x+\tau }^{j}$, and 0 otherwise. Additionally, all of the operations among the position indices ${\left(\cdot \right)}_{x+\tau }$ are performed modulo ι. In addition, $H_{g^{i},g^{j}}\left(\tau \right)$ is called the periodic Hamming cross-correlation function when i≠j, while it is called the periodic Hamming autocorrelation function when i=j.

The maximum periodic Hamming cross-correlation $H_{cm}\left(G\right)$, the maximum periodic Hamming autocorrelation $H_{am}\left(G\right)$, and the maximum PHC $H_{m}\left(G\right)$ of *G* are, respectively, defined as follows:

$$\begin{array}{l} H_{cm}\left(G\right)=\max\left\{H_{g^{i},g^{j}}\left(\tau \right),0\leq \tau <\iota ,0\leq i\neq j<\eta \right\},\\ H_{am}\left(G\right)=\max\left\{H_{g^{i},g^{i}}\left(\tau \right),1\leq \tau <\iota ,0\leq i<\eta \right\},\\ H_{m}\left(G\right)=\max\left\{H_{am}\left(G\right),H_{cm}\left(G\right)\right\}. \end{array}$$

In 2004, Peng et al. [5] derived the following lower bound on the maximum PHC of an FHS set, which is also known as Peng–Fan bound.

**Lemma** **1** ([5]: Peng–Fan bound)**.** *Let G be an FHS set with η frequency-hopping sequences of length ι over F with size ρ. Then,*

(1) $$\begin{array}{c} H_{m}\left(G\right)\geq \left\lceil \frac{2I\iota \eta -(I+1)I\rho }{(\iota \eta -1)\eta }\right\rceil , \end{array}$$

*where $I=\left\lfloor \frac{\iota \eta }{\rho }\right\rfloor$.*

For the FHS set *G*, let integers $H_{az}>0,H_{cz}>0$. Then, the periodic Hamming cross-correlation LHZ $\iota_{cz}$, the periodic Hamming autocorrelation LHZ $\iota_{az}$, the PHC LHZ $\iota_{z}$, and the maximum PHC $H_{zm}\left(G\right)$ within the LHZ of *G* are, respectively, defined as

$$\begin{array}{c} \iota_{cz}=\max_{0\leq i\neq j<\eta }\left\{Z|H_{g^{i},g^{j}}\left(\tau \right)\leq H_{cz},0\leq \tau \leq Z\right\},\\ \iota_{az}=\max_{0\leq i<\eta }\left\{Z|H_{g^{i},g^{i}}\left(\tau \right)\leq H_{az},0<\tau \leq Z\right\},\\ \iota_{z}=\min\left\{\iota_{az},\iota_{cz}\right\},\\ H_{zm}\left(G\right)=\max\left\{H_{az},H_{cz}\right\}. \end{array}$$

Specially, if $\iota_{z}=\iota -1$, *G* can be viewed as a conventional FHS set, and its maximum PHC is denoted as $H_{m}\left(G\right)$.

In 2006, Peng et al. [4] derived the following lower bound on the maximum PHC of an LHZ-FHS set, which is also known as Peng–Fan–Lee bound.

**Lemma** **2** ([4]: Peng–Fan–Lee bound)**.** *Let G be an FHS set with η frequency-hopping sequences of length ι over F with size ρ, and $\iota_{z}$ be its LHZ with regard to $H_{zm}\left(G\right)$. Then, for any positive integer $Z,0\leq Z\leq \iota_{z}$,*

(2) $$\begin{array}{c} H_{zm}\left(G\right)\geq \left\lceil \frac{\left(Z+1\right)\left(2I\iota \eta +\iota \eta -I\rho -I^{2}\rho \right)-\eta \iota^{2}}{(\eta Z+\eta -1)\iota \eta }\right\rceil , \end{array}$$

*where $I=\left\lfloor \frac{\iota \eta }{\rho }\right\rfloor$.*

Obviously, the bound (1) is an exceptional case of the bound (2) when the parameter *Z* in the bound (2) equals ι−1.

Assume that we have an FHS set *G*, consisting of η frequency-hopping sequences with length ι over frequency slot set *F* with size ρ, and having maximum PHC $H_{zm}\left(G\right)$ within the LHZ $\iota_{z}$. For the sake of simplicity, we denote *G* as an $(\iota ,\eta ,\rho ,\iota_{z}$, $H_{zm}\left(G\right))$ LHZ-FHS set. Specially, if $\iota_{z}=\iota -1$, we denote *G* as an $\left(\iota ,\eta ,\rho ,H_{m}\left(G\right)\right)$ FHS set.

**Definition** **1.** *For the $\left(\iota ,\eta ,\rho ,H_{m}\left(G\right)\right)$ FHS set G, if its parameters can meet the equality in Bound (1), it is an optimal FHS set. Similarly, for the $(\iota ,\eta ,\rho ,\iota_{z}$, $H_{zm}\left(G\right))$ LHZ-FHS set G, it is said to be an optimal LHZ-FHS set if the equality in Bound (2) is achieved, while it is said to be a near-optimal LHZ-FHS set if the right side of Bound (2) equals $H_{zm}\left(G\right)-1$.*

## 3. General Construction of New LHZ-FHS Sets via Interleaving Technique

The interleaved structure of sequences was proposed by Gong in [26,27]. Afterwards, some FHS sets were obtained via the interleaving technique [13,15,16,25]. In this paper, we will also introduce a general construction to design new families of LHZ-FHS sets through the interleaving technique. Some basics about the interleaved structure will be introduced firstly.

Let $g=[g_{x},0\leq x<\iota ]$ be an FHS of length ι over the frequency slot set *F* with size ρ, $Z_{\iota }$ be the residual class ring of integers modulo ι, and $w=[w_{y},0\leq y<\lambda ]$ be a shift sequence of length λ over $Z_{\iota }$. Then, an ι×λ matrix can be formed by placing *g* and *w* as follows:

(3) $$\begin{array}{ccc} U & = & \left(\begin{array}{cccc} g_{0+w_{0}} & g_{0+w_{1}} & \ldots & g_{0+w_{\lambda -1}}\\ g_{1+w_{0}} & g_{1+w_{1}} & \ldots & g_{1+w_{\lambda -1}}\\ \vdots & \vdots & \ddots & \vdots \\ g_{\iota -1+w_{0}} & g_{\iota -1+w_{1}} & \ldots & g_{\iota -1+w_{\lambda -1}} \end{array}\right)\\ & = & \left(\begin{array}{cccc} u_{0} & u_{1} & \ldots & u_{\lambda -1}\\ u_{\lambda } & u_{\lambda +1} & \ldots & u_{2\lambda -1}\\ \vdots & \vdots & \ddots & \vdots \\ u_{\lambda (\iota -1)} & u_{\lambda (\iota -1)+1} & \ldots & u_{\iota \lambda -1} \end{array}\right). \end{array}$$

By reading the elements in *U* row by row, we obtain a new sequence *u* of period ιλ over *F*. Conventionally, *u* is called the interleaved sequence, and *g* or *w* is referred to its base sequence or shift sequence, respectively. For short, we denote the interleaved sequence *u* as:

$$\begin{array}{c} u=\mathrm{Intl}(L^{w_{0}}g,L^{w_{1}}g,\cdots ,L^{w_{\lambda -1}}g), \end{array}$$

where Intl is the interleaving operator, and L is the cyclical left shift operator, such that $L^{i}g=\left[g_{i},\cdots ,g_{\iota -1},g_{0},\cdots ,g_{i-1}\right]$.

Let $t=[t_{y},0\leq y<\lambda ]$ be another shift sequence of length λ over $Z_{\iota }$. Then, through the base FHS *g* and the shift sequence *t*, we can obtain another interleaved sequence *v* as

$$\begin{array}{c} v=\mathrm{Intl}(L^{t_{0}}g,L^{t_{1}}g,\cdots ,L^{t_{\lambda -1}}g). \end{array}$$

For the interleaved sequence *v* and the time delay $\tau =\lambda \tau_{1}+\tau_{2},0\leq \tau_{1}<\iota ,0\leq \tau_{2}<\lambda$, we can represent the shift sequence $L^{\tau }v$ of *v* as

$$\begin{array}{c} V=\left(\begin{array}{cccccc} g_{t_{\tau_{2}}+\tau_{1}} & \ldots & g_{t_{\lambda -1}+\tau_{1}} & g_{t_{0}+\tau_{1}+1} & \ldots & g_{t_{\tau_{2}-1}+\tau_{1}+1}\\ g_{t_{\tau_{2}}+\tau_{1}+1} & \ldots & g_{t_{\lambda -1}+\tau_{1}+1} & g_{t_{0}+\tau_{1}+2} & \ldots & g_{t_{\tau_{2}-1}+\tau_{1}+2}\\ \vdots & \ddots & \vdots & \vdots & \ddots & \vdots \\ g_{t_{\tau_{2}}+\tau_{1}-1} & \ldots & g_{t_{\lambda -1}+\tau_{1}-1} & g_{t_{0}+\tau_{1}} & \ldots & g_{t_{\tau_{2}-1}+\tau_{1}} \end{array}\right). \end{array}$$

Obviously, $L^{\tau }v$ is also an interleaved sequence, and can be written as

$$\begin{array}{c} L^{\tau }v=\mathrm{Intl}\left(L^{t_{\tau_{2}}+\tau_{1}}g,\cdots ,L^{t_{\lambda -1}+\tau_{1}}g,L^{t_{0}+\tau_{1}+1}g,\cdots ,L^{t_{\tau_{2}-1}+\tau_{1}+1}g\right). \end{array}$$

Therefore, the calculation of the PHC function between *u* and *v* becomes the summation of the inner products between the pairwise columns in the matrices *U* and *V*. Namely,

(4) $$\begin{array}{ccc} H_{u,v}\left(\tau \right) & = & \sum_{i=0}^{\lambda -\tau_{2}-1}H_{g,g}\left(t_{i+\tau_{2}}+\tau_{1}-w_{i}\right)+\sum_{i=\lambda -\tau_{2}}^{\lambda -1}H_{g,g}\left(t_{i+\tau_{2}-\lambda }+\tau_{1}+1-w_{i}\right). \end{array}$$

Let

$$\begin{array}{c} d_{i,\tau_{2}}^{\left(w,t\right)}=\left\{\begin{array}{cc} w_{i}-t_{i+\tau_{2}}, & \;0\leq i\leq \lambda -1-\tau_{2}\\ w_{i}-t_{i+\tau_{2}-\lambda }-1, & \;\lambda -\tau_{2}\leq i\leq \lambda -1 \end{array}\right., \end{array}$$

where $d_{i,\tau_{2}}^{\left(w,t\right)}$ should be calculated modulo ι. We can rewrite the Formula (4) as

(5) $$\begin{array}{c} H_{u,v}\left(\tau \right)=\sum_{i=0}^{\lambda -1}H_{g,g}\left(\tau_{1}-d_{i,\tau_{2}}^{\left(w,t\right)}\right). \end{array}$$

**Lemma** **3** ([13])**.** *With the notations as above, for the interleaved sequence u and v, if $\tau_{1}<\min_{0\leq i,\tau_{2}<\lambda }\left\{d_{i,\tau_{2}}^{\left(w,t\right)}\right\}$, we have*

$$\begin{array}{c} H_{u,v}\left(\tau \right)\leq \lambda H_{am}\left(g\right), \end{array}$$

*where $\tau =\lambda \tau_{1}+\tau_{2},0\leq \tau_{1}<\iota ,0\leq \tau_{2}<\lambda$, and $H_{am}\left(g\right)$ is the maximum periodic Hamming autocorrelation of g.*

Different from the constructions in [13], we have the following general construction to design LHZ-FHS sets with just one shift sequence.

**General construction.**

*Step 1:* choose an $\left(\iota ,\eta ,\rho ,H_{m}\left(G\right)\right)$ FHS set $G=\{g^{j}=\left[g_{x}^{j},0\leq x<\iota \right],0\leq j<\eta \}$ as the base FHS set.

*Step 2:* select a positive integer λ with λ≥2 and generate a shift sequence $w=[w_{y},0\leq y<\lambda ]$.

*Step 3:* construct a new FHS set $A=\{a^{j}=\left[a_{x}^{j},0\leq x<\iota \lambda \right],0\leq j<\eta \}$ as follows:

$$\begin{array}{c} a^{j}=\mathrm{Intl}\left(L^{w_{0}}g^{j},L^{w_{1}}g^{j},\cdots ,L^{w_{\lambda -1}}g^{j}\right). \end{array}$$

**Theorem** **1.** *The FHS set A in the general construction is an $\left(\iota \lambda ,\eta ,\rho ,\iota_{z},\lambda H_{m}\left(G\right)\right)$ LHZ-FHS set, where*

$$\begin{array}{c} \iota_{z}=\min_{0\leq i,\tau_{2}<\lambda }\left\{\lambda d_{i,\tau_{2}}^{\left(w,w\right)}+\tau_{2}\right\}-1. \end{array}$$

**Proof.**  Obviously, there are η sequences of length ιλ in the FHS set *A*. Let $H_{am}\left(G\right)$ and $H_{cm}\left(G\right)$ represent the maximum periodic Hamming autocorrelation and the maximum periodic Hamming cross-correlation of *G*, respectively. For any $a^{i},a^{j}\in A,0\leq i,j<\eta$, their PHC function $H_{a^{i},a^{j}}\left(\tau \right)$ at relative time delay τ can be given in the following two cases.

*Case 1:* i=j,0<τ<ιλ. According to Lemma 3,

$$\begin{array}{c} H_{a^{i},a^{i}}\left(\tau \right)=\sum_{i=0}^{\lambda -1}H_{g^{i},g^{i}}\left(\tau_{1}-d_{i,\tau_{2}}^{\left(w,w\right)}\right)\leq \lambda H_{am}\left(G\right) \end{array}$$

as long as $0<\tau_{1}<\min_{0\leq i,\tau_{2}<\lambda }\left\{d_{i,\tau_{2}}^{\left(w,w\right)}\right\}$.

*Case 2:* i≠j,0≤τ<ιλ. Similarly to Expression (5), we can easily have

$$\begin{array}{c} H_{a^{i},a^{j}}\left(\tau \right)=\sum_{i=0}^{\lambda -1}H_{g^{i},g^{j}}\left(\tau_{1}-d_{i,\tau_{2}}^{\left(w,w\right)}\right)\leq \lambda H_{cm}\left(G\right). \end{array}$$

In brief, the maximum PHC of *A* within the LHZ $\iota_{z}$ can be given by

$$\begin{array}{c} H_{zm}\left(A\right)=\max\left\{\lambda H_{am}\left(G\right),\lambda H_{cm}\left(G\right)\right\}=\lambda H_{m}\left(G\right), \end{array}$$

where $\iota_{z}=\min_{0\leq i,\tau_{2}<\lambda }\left\{\lambda d_{i,\tau_{2}}^{\left(w,w\right)}+\tau_{2}\right\}-1$. This completes the proof. □

**Remark** **1.** *Compared with the constructions in [13], in our general construction, there is no need to satisfy the condition that λ and ι should be coprime. Additionally, as only one shift sequence rather than a shift sequence set is employed, we can obtain a bigger LHZ value.*

## 4. Two New Classes of Optimal or Near-Optimal LHZ-FHS Sets

In this section, through two new different shift sequences, we will introduce two classes of optimal or near-optimal LHZ-FHS sets with new parameters. Comparing to the constructions in [13] or [15], those in this paper have different parameter constrains and shift sequences or different base FHS sets. For simplicity, we use RSLH[ι,η,ρ, $\iota_{z}]=\left\lceil \frac{\left(\iota_{z}+1\right)\left(\left(2I+1\right)\iota \eta -I^{2}\rho -I\rho \right)-\eta \iota^{2}}{(\eta \iota_{z}+\eta -1)\iota \eta }\right\rceil$ to represent the right side of the bound (2) in the rest of this paper.

**Construction 1.**

*Step 1:* choose an optimal $\left(\iota ,\eta ,\rho ,H_{m}\left(G\right)\right)$ FHS set $G=\{g^{j}=\left[g_{x}^{j},0\leq x<\iota \right]$, 0≤j<η} as the base FHS set.

*Step 2:* Choose two positive integers κ and λ satisfying κλ=ι and λ≥2. Let δ be an integer with −λ<δ<λ and gcd(δ,ι)=1. Then, generate a shift sequence $w=[w_{y},0\leq y<\lambda ]$ as follows:

$$\begin{array}{c} w=\left[w_{0},w_{1}\cdots ,w_{\lambda -1}\right]=\left[\left(\lambda -1\right)\kappa \delta^{-1},\left(\lambda -2\right)\kappa \delta^{-1},\cdots ,0\right], \end{array}$$

where $\delta^{-1}$ is the inverse element of δ modulo ι, and $w_{y},\;0\leq y<\lambda$ should be calculated modulo ι.

*Step 3:* construct a new FHS set $B=\{b^{j}=\left[b_{x}^{j},0\leq x<\iota \lambda \right],0\leq j<\eta \}$ as follows:

$$\begin{array}{c} b^{j}=\mathrm{Intl}\left(L^{w_{0}}g^{j},L^{w_{1}}g^{j},\cdots ,L^{w_{\lambda -1}}g^{j}\right). \end{array}$$

**Theorem** **2.** *The FHS set B in Construction 1 is an $(\iota \lambda ,\eta ,\rho ,\iota_{z1}$, $\lambda H_{m}\left(G\right))$ LHZ-FHS set, where*

$$\begin{array}{c} \iota_{z1}=\left\{\begin{array}{cc} \lambda (\kappa -1)+\delta -1, & \mathrm{if}\;0<\delta <\lambda \\ \lambda \kappa +\delta -1, & \mathrm{if}\;-\lambda <\delta <0 \end{array}\right.. \end{array}$$

*Moreover, if*

(6) $$\begin{array}{c} \mathrm{RSLH}\left[\iota \lambda ,\eta ,\rho ,\iota_{z1}\right]=\lambda \mathrm{RSLH}\left[\iota ,\eta ,\rho ,\iota -1\right] \end{array}$$

*or*

(7) $$\begin{array}{c} \mathrm{RSLH}\left[\iota \lambda ,\eta ,\rho ,\iota_{z1}\right]=\lambda \mathrm{RSLH}\left[\iota ,\eta ,\rho ,\iota -1\right]-1, \end{array}$$

*then B is an optimal or a near-optimal LHZ-FHS set.*

**Proof.**  According to Theorem 1, *B* is an LHZ-FHS set with parameters $\left(\iota \lambda ,\eta ,\rho ,\iota_{z1},\lambda H_{m}\left(G\right)\right)$, where

$$\begin{array}{c} L_{z1}=\min_{0\leq i<\lambda ,0<\tau_{2}<\lambda }\left\{\lambda d_{i,\tau_{2}}^{\left(w,w\right)}+\tau_{2}\right\}-1. \end{array}$$

Apparently, we have

$$\begin{array}{ccc} d_{i,\tau_{2}}^{\left(w,w\right)} & = & \left\{\begin{array}{cc} w_{i}-w_{i+\tau_{2}}, & \;0\leq i\leq \lambda -1-\tau_{2}\\ w_{i}-w_{i+\tau_{2}-\lambda }-1, & \;\lambda -\tau_{2}\leq i\leq \lambda -1 \end{array}\right.\\ & = & \left\{\begin{array}{cc} \tau_{2}\kappa \delta^{-1}, & \;0\leq i\leq \lambda -1-\tau_{2}\\ \left(\tau_{2}-\lambda \right)\kappa \delta^{-1}-1, & \;\lambda -\tau_{2}\leq i\leq \lambda -1 \end{array}\right.. \end{array}$$

On the other hand, as κλ=ι and $d_{i,\tau_{2}}^{\left(w,w\right)}$ should be calculated modulo ι, we have

$$\begin{array}{ccc} d_{i,\tau_{2}}^{\left(w,w\right)} & = & \left\{\begin{array}{cc} \tau_{2}\kappa \delta^{-1}, & \;0\leq i\leq \lambda -1-\tau_{2}\\ \tau_{2}\kappa \delta^{-1}-1, & \;\lambda -\tau_{2}\leq i\leq \lambda -1 \end{array}\right.. \end{array}$$

Thus, when $\tau_{2}\equiv \delta$ (mod λ), $d_{i,\tau_{2}}^{\left(w,w\right)}$ reaches its minimum value, κ−1. Additionally, as $0\leq \tau_{2}<\lambda$, we have the following conclusion:

$$\begin{array}{c} L_{z1}=\left\{\begin{array}{cc} \lambda (\kappa -1)+\delta -1, & \mathrm{if}\;0<\delta <\lambda \\ \lambda \kappa +\delta -1, & \mathrm{if}\;-\lambda <\delta <0 \end{array}\right.. \end{array}$$

Put the parameters of *B* into the bound (2). We have

$$\begin{array}{c} H_{zm}\left(B\right)\geq \mathrm{RSLH}\left[\iota \lambda ,\eta ,\rho ,\iota_{z1}\right]. \end{array}$$

Furthermore, as *G* is an optimal FHS set with respect to the bound (1), we have

$$\begin{array}{c} H_{m}\left(G\right)=\mathrm{RSLH}\left[\iota ,\eta ,\rho ,\iota -1\right]. \end{array}$$

Then, according to Definition 1, the conditions under which the LHZ-FHS set *B* can be optimal or near-optimal with respect to Bound (2) should be

$$\begin{array}{c} \mathrm{RSLH}\left[\iota \lambda ,\eta ,\rho ,\iota_{z1}\right]=\lambda \mathrm{RSLH}\left[\iota ,\eta ,\rho ,\iota -1\right] \end{array}$$

or

$$\begin{array}{c} \mathrm{RSLH}\left[\iota \lambda ,\eta ,\rho ,\iota_{z1}\right]=\lambda \mathrm{RSLH}\left[\iota ,\eta ,\rho ,\iota -1\right]-1. \end{array}$$

This completes the proof. □

**Example** **1.** *Choose an optimal (25,5,5,5) base FHS set $G=\{g^{j}=\left[g_{x}^{j},0\leq x<25\right],0\leq j<5\}$ as*

$$\begin{array}{ccc} & & g^{0}=\left[1,1,1,1,1,1,0,2,3,4,1,2,4,0,3,1,3,0,4,2,1,4,3,2,0\right],\\ & & g^{1}=\left[0,0,4,4,0,0,2,0,2,1,0,3,3,1,4,0,4,1,3,3,0,1,2,0,2\right],\\ & & g^{2}=\left[2,2,3,3,2,2,3,1,0,0,2,4,2,4,1,2,1,4,2,4,2,0,0,1,3\right],\\ & & g^{3}=\left[3,3,2,2,3,3,4,4,1,2,3,1,0,3,0,3,0,3,0,1,3,2,1,4,4\right],\\ & & g^{4}=\left[4,4,0,0,4,4,1,3,4,3,4,0,1,2,2,4,2,2,1,0,4,3,4,3,1\right]. \end{array}$$

*Let κ=5,λ=5 and δ=3. Based on Construction 1, we can generate a shift sequence $w=[w_{y},0\leq y<5]$ as*

$$\begin{array}{c} w=[15,5,20,10,0]. \end{array}$$

*Then, according to Construction 1, we can obtain a new FHS set $B=\{b^{j}=\left[b_{x}^{j},0\leq x<125\right]$, 0≤j<5} as*

$$\begin{array}{ccc} b^{0} & = & [1,1,1,1,1,3,0,4,2,1,0,2,3,4,1,4,3,2,0,1,2,4,0,3,1,1,1,1,1,1,4,2,1,3,\\ & & 0,3,4,1,0,2,2,0,1,4,3,0,3,1,2,4,1,1,1,1,1,1,3,0,4,2,1,0,2,3,4,1,\cdots ],\\ b^{1} & = & [0,0,0,0,0,4,2,1,3,0,1,0,2,3,4,3,2,0,1,4,3,1,2,4,0,0,0,0,0,0,1,3,0,4,\\ & & 2,2,3,4,1,0,0,1,4,3,2,2,4,0,3,1,0,0,0,0,0,0,4,2,1,3,4,1,0,2,3,4,\cdots ],\\ b^{2} & = & [2,2,2,2,2,1,3,0,4,2,4,1,0,2,3,2,0,1,4,3,4,0,3,1,2,2,2,2,2,2,0,4,2,1,\\ & & 3,0,2,3,4,1,1,4,3,2,0,3,1,2,4,0,2,2,2,2,2,2,1,3,0,4,3,4,1,0,2,3,\cdots ],\\ b^{3} & = & [3,3,3,3,3,0,4,2,1,3,3,4,1,0,2,0,1,4,3,2,1,2,4,0,3,3,3,3,3,3,2,1,3,0,\\ & & 4,1,0,2,3,4,4,3,2,0,1,4,0,3,1,2,3,3,3,3,3,3,0,4,2,1,2,3,4,1,0,2,\cdots ],\\ b^{4} & = & [4,4,4,4,4,2,1,3,0,4,2,3,4,1,0,1,4,3,2,0,0,3,1,2,4,4,4,4,4,4,3,0,4,2,\\ & & 1,4,1,0,2,3,3,2,0,1,4,1,2,4,0,3,4,4,4,4,4,4,2,1,3,0,0,2,3,4,1,0,\cdots ]. \end{array}$$

**Figure 1:**
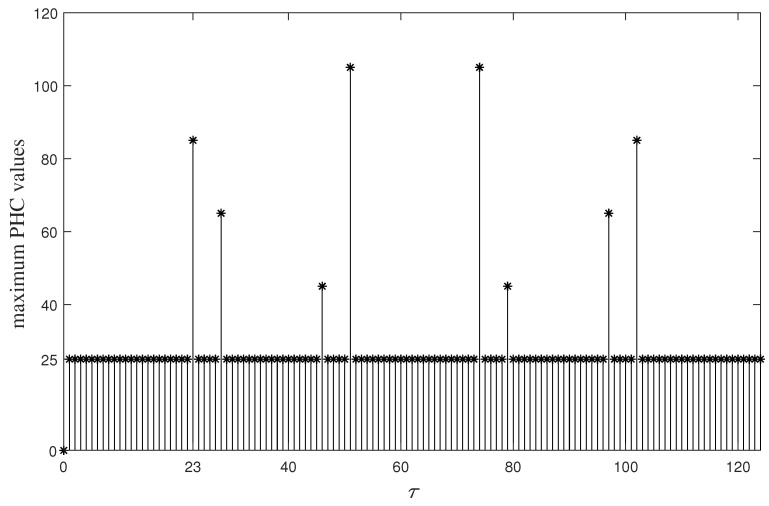
The maximum PHC values of FHS set *B* under different time delays (τ).

*Figure 1 illustrates the maximum PHC values of FHS set B under different time delays τ,0≤τ<125. From Figure 1, it can be seen that we always have*

$$\begin{array}{c} H_{b^{i},b^{j}}\left(\tau \right)\leq 25 \end{array}$$

*for any $b^{i},b^{j}\in B,0\leq i,j<5$ and τ≤22. Therefore, B is a (125,5,5,22,25) LHZ-FHS set. Put the parameters of B and G into (6). It is easy to verify that both the left and right sides of (6) equal 25. Then, according to Theorem 2, B is an optimal LHZ-FHS set.*

**Figure 2:**
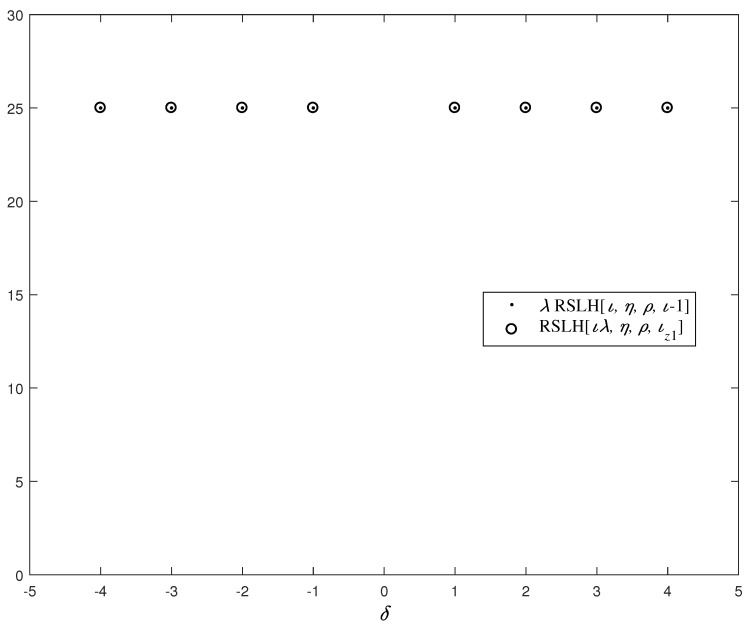
The PHC performance of the LHZ-FHS set *B*.

*Furthermore, according to Construction 1, the value of δ can be −4, −3, −2, −1, 1, 2, 3 or 4. Through computer experiments, along with changes in δ, we analyze the corresponding $\mathrm{RSLH}[\iota \lambda ,\eta ,\rho ,\iota_{z1}]$ and λRSLH[ι,η,ρ,ι−1], as shown in Figure 2, where ι=25, η=5, ρ=5 and λ=5. On the basis of Figure 2 and Theorem 2, for all the possible δ, B is always an optimal LHZ-FHS set.*

**Construction 2.**

*Step 1:* choose an optimal $\left(\iota ,\eta ,\rho ,H_{m}\left(G\right)\right)$ FHS set $G=\{g^{j}=\left[g_{x}^{j},0\leq x<\iota \right]$, 0≤j<η} as the base FHS set.

*Step 2:* Choose an integer κ satisfying $2\leq \kappa \leq \left\lfloor \frac{\iota }{2}\right\rfloor$. Let $\lambda =\left\lfloor \frac{\iota }{\kappa }\right\rfloor$. Then, generate a shift sequence $t=[t_{y},0\leq y<\lambda ]$ as follows:

$$\begin{array}{c} t=\left[t_{0},t_{1},\cdots ,t_{\lambda -1}\right]=\left[\left(\lambda -1\right)\kappa ,\left(\lambda -2\right)\kappa ,\cdots ,0\right]. \end{array}$$

*Step 3:* construct a new FHS set $C=\{c^{j}=\left[c_{x}^{j},0\leq x<\iota \lambda \right],0\leq j<\eta \}$ as follows:

$$\begin{array}{c} c^{j}=\mathrm{Intl}\left(L^{t_{0}}g^{j},L^{t_{1}}g^{j},\cdots ,L^{t_{\lambda -1}}g^{j}\right). \end{array}$$

**Theorem** **3.** *The FHS set C in Construction 2 is an $\left(\iota \lambda ,\eta ,\rho ,\iota_{z2},\lambda H_{m}\left(G\right)\right)$ LHZ-FHS set, where*

$$\begin{array}{c} \iota_{z2}=\left\{\begin{array}{cc} \lambda (\kappa -1), & \mathrm{if}\;\kappa |\iota \\ \lambda \kappa , & \mathrm{if}\;\kappa ∤\iota \end{array}\right.. \end{array}$$

*Moreover, if*

(8) $$\begin{array}{c} \mathrm{RSLH}\left[\iota \lambda ,\eta ,\rho ,\iota_{z2}\right]=\lambda \mathrm{RSLH}\left[\iota ,\eta ,\rho ,\iota -1\right] \end{array}$$

*or*

(9) $$\begin{array}{c} \mathrm{RSLH}\left[\iota \lambda ,\eta ,\rho ,\iota_{z2}\right]=\lambda \mathrm{RSLH}\left[\iota ,\eta ,\rho ,\iota -1\right]-1, \end{array}$$

*then C is an optimal or a near-optimal LHZ-FHS set.*

**Proof.**  Here, we are only concerned with the value of $\iota_{z2}$, and the rest of the proof is similar to the proof of Theorem 2. Obviously, we have

$$\begin{array}{ccc} d_{i,\tau_{2}}^{\left(t,t\right)} & = & \left\{\begin{array}{cc} t_{i}-t_{i+\tau_{2}}, & \;0\leq i\leq \lambda -1-\tau_{2}\\ t_{i}-t_{i+\tau_{2}-\lambda }-1, & \;\lambda -\tau_{2}\leq i\leq \lambda -1 \end{array}\right.\\ & = & \left\{\begin{array}{cc} \tau_{2}\kappa , & \;0\leq i\leq \lambda -1-\tau_{2}\\ (\tau_{2}-\lambda )\kappa -1, & \;\lambda -\tau_{2}\leq i\leq \lambda -1 \end{array}\right.. \end{array}$$

*Case 1:* κ∣ι. We have $\lambda =\left\lfloor \frac{\iota }{\kappa }\right\rfloor =\frac{\iota }{\kappa }$. Namely, λκ=ι. Then,

$$\begin{array}{c} d_{i,\tau_{2}}^{\left(t,t\right)}=\left\{\begin{array}{cc} \tau_{2}\kappa , & \;0\leq i\leq \lambda -1-\tau_{2}\\ \tau_{2}\kappa -1, & \;\lambda -\tau_{2}\leq i\leq \lambda -1 \end{array}\right.. \end{array}$$

Thus, when $\tau_{2}=1$, $d_{i,\tau_{2}}^{\left(t,t\right)}$ reaches its minimum value, κ−1. Thus, we have

$$\begin{array}{c} \iota_{z2}=\lambda \left(\kappa -1\right). \end{array}$$

*Case 2:* κ∤ι. We have $\lambda =\left\lfloor \frac{\iota }{\kappa }\right\rfloor <\frac{\iota }{\kappa }$. Let ι=λκ+k,1≤k<κ. Then,

$$\begin{array}{c} d_{i,\tau_{2}}^{\left(t,t\right)}=\left\{\begin{array}{cc} \tau_{2}\kappa , & \;0\leq i\leq \lambda -1-\tau_{2}\\ \tau_{2}\kappa +k-1, & \;\lambda -\tau_{2}\leq i\leq \lambda -1 \end{array}\right.. \end{array}$$

As k≥1, we always have $\tau_{2}\kappa +k-1\geq \tau_{2}\kappa$. Therefore, when $\tau_{2}=1$, $d_{i,\tau_{2}}^{\left(t,t\right)}$ reaches its minimum value, κ. Thus, we have

$$\begin{array}{c} \iota_{z2}=\lambda \kappa . \end{array}$$

In brief, we have the following conclusion:

$$\begin{array}{c} \iota_{z2}=\left\{\begin{array}{cc} \lambda (\kappa -1), & \mathrm{if}\;\kappa |\iota \\ \lambda \kappa , & \mathrm{if}\;\kappa ∤\iota \end{array}\right.. \end{array}$$

This completes the proof. □

**Example** **2.** *Choose an optimal (14,4,8,2) base FHS set $G=\{g^{j}=\left[g_{x}^{j},0\leq x<14\right],0\leq j<4\}$ as*

$$\begin{array}{ccc} & & g^{0}=\left[0,7,1,3,2,6,3,1,4,5,5,4,6,2\right],\\ & & g^{1}=\left[3,6,7,4,4,7,0,5,2,1,6,3,5,0\right],\\ & & g^{2}=\left[1,5,0,2,5,1,7,6,6,7,2,0,4,3\right],\\ & & g^{3}=\left[4,2,6,5,3,0,2,4,0,3,7,1,1,7\right]. \end{array}$$

*Let κ=5. Then, we have $\lambda =\left\lfloor \frac{14}{\kappa }\right\rfloor =2$ and the shift sequence $t=[t_{y},0\leq y<2]$ as*

$$\begin{array}{c} t=[5,0]. \end{array}$$

*According to Construction 2, we can obtain a new FHS set $C=\{c^{j}=\left[c_{x}^{j},0\leq x<28\right]$, 0≤j<4} as*

$$\begin{array}{ccc} & & c^{0}=\left[6,0,3,7,1,1,4,3,5,2,5,6,4,3,6,1,2,4,0,5,7,5,1,4,3,6,2,2\right],\\ & & c^{1}=\left[7,3,0,6,5,7,2,4,1,4,6,7,3,0,5,5,0,2,3,1,6,6,7,3,4,5,4,0\right],\\ & & c^{2}=\left[1,1,7,5,6,0,6,2,7,5,2,1,0,7,4,6,3,6,1,7,5,2,0,0,2,4,5,3\right],\\ & & c^{3}=\left[0,4,2,2,4,6,0,5,3,3,7,0,1,2,1,4,7,0,4,3,2,7,6,1,5,1,3,7\right]. \end{array}$$

**Figure 3:**
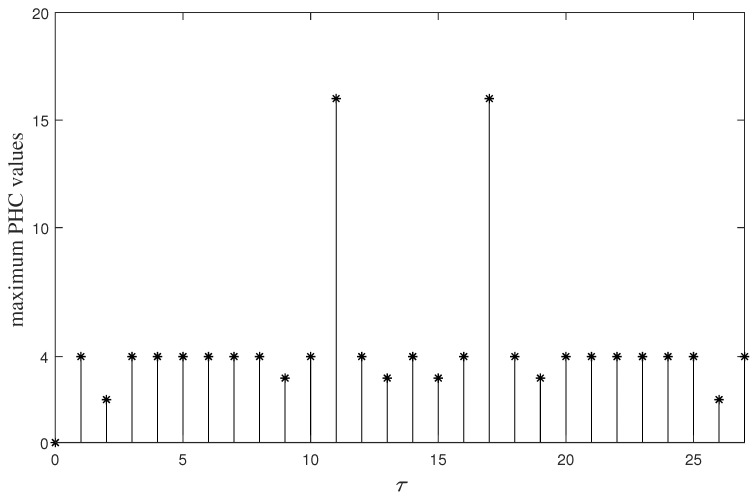
The maximum PHC values of FHS set *C* under different time delay τ.

*Figure 3 illustrates the maximum PHC values of FHS set C under different time delay τ,0≤τ<28. From Figure 3, it can be seen that we always have*

$$\begin{array}{c} H_{c^{i},c^{j}}\left(\tau \right)\leq 4 \end{array}$$

*for any $c^{i},c^{j}\in C,0\leq i,j<4$ and τ≤10. Therefore, C is a (28,4,8,10,4) LHZ-FHS set. Put the parameters of C and G into (9). It is easy to verify that both the left and right sides of (9) equal 3. Then, on the basis of Theorem 3, C is a near-optimal LHZ-FHS set in this situation.*

**Figure 4:**
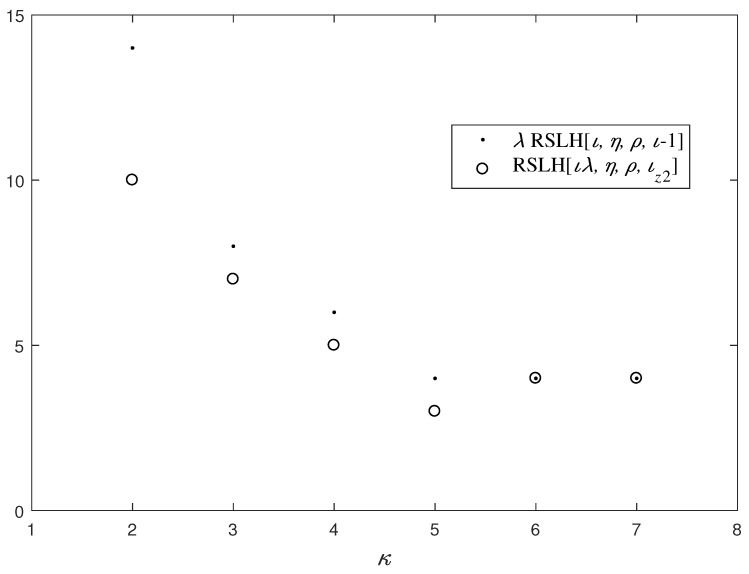
The PHC performance of the LHZ-FHS set *C*.

*Furthermore, based on Construction 2, we have 2≤κ≤7. Through computer experiments, as the value of κ varies from 2 to 7, we analyze the corresponding $\mathrm{RSLH}[\iota \lambda ,\eta ,\rho ,\iota_{z2}]$ and λRSLH[ι,η,ρ,ι−1], as shown in Figure 4, where ι=14, η=4, ρ=8 and $\lambda =\left\lfloor \frac{14}{\kappa }\right\rfloor$. According to Figure 4 and Theorem 3, C is a near-optimal LHZ-FHS set if 3≤κ≤5, while it is an optimal LHZ-FHS set if 6≤κ≤7.*

**Remark** **2.** *To better accommodate different possible base sequence lengths, the relationships among ι, κ, and λ in Construction 1 and Construction 2 are generally distinct. Especially, if κ∣ι and δ=1, then Construction 1 is same as Construction 2.*

## 5. Conclusions

In this paper, we had a general construction to design new families of LHZ-FHS sets through interleaving technique, based on which we introduced two new classes of LHZ-FHS sets. We also presented the requirements for our new LHZ-FHS sets to reach the optimality or near-optimality with regard to the Peng–Fan–Lee bound. Furthermore, provided that the base FHS set is selected, we can analyze the performances of all the newly constructed LHZ-FHS sets, which does bring convenience for us to choose the desirable value of κ, λ, or δ. Remarkably, our constructed LHZ-FHS sets have new parameters not included in the relevant literature (see Table 1), and so can be useful in QS-FHMA systems to minimize the MI.

## Figures and Tables

**Table 1:** Comparison of parameters of different LHZ-FHS sets.

Parameters $\left(\iota ,\eta ,\rho ,\iota_{z},H_{\mathrm{zm}}\right)$	Constraints	Reference
$\left(j_{1}\left(q^{n}-1\right),i_{1},q,Z_{1},j_{1}\left(q^{n-1}-1\right)\right)$	n≥1, $q^{n}-1=i_{1}\left(Z_{1}+1\right)$, $\gcd(j_{1},q^{n}-1)=1$, $j_{1}<i_{1}$	[12]
$\left(j_{2}\left(q^{n}-1\right),i_{2}q^{k},q^{k},Z_{2},j_{2}\left(q^{n-k}-1\right)\right)$	n≥1, 0<k≤n, $q^{n}-1=i_{2}\left(Z_{2}+1\right)$, $j_{2}\equiv 1\;\mathrm{mod}\;(Z_{2}+1)$, $2\leq j_{2}<\frac{q^{k}\left(q^{n}-2\right)}{q-1}$	[13]
$\left(j_{3}\frac{q^{n}-1}{l},i_{3}l,q,Z_{3},j_{3}\frac{q^{n-1}-1}{l}\right)$	n≥1, $\frac{q^{n}-1}{l}=i_{3}\left(Z_{3}+1\right)$, gcd(l,n)=1, $j_{3}\equiv 1\;\mathrm{mod}\;\left(Z_{3}+1\right)$, $2\leq j_{3}<\frac{lq(q^{n}-2)}{q-1}$	[13]
$(p^{2}\left(q_{1}-1\right)\left(q_{2}-1\right),pq_{1}q_{2},pq_{1}q_{2}$, $\min\{p^{2}-1,q_{1}-2,q_{2}-2\},p)$	$\gcd(p,q_{1}-1,q_{2}-1)=1$, $3p<\min\{q_{1}-1,q_{2}-1\}$	[14]
$(k_{1}k_{2}\left(q_{1}-1\right)\left(q_{2}-1\right),\frac{\left(q_{1}-1\right)\left(q_{2}-1\right)}{k_{1}k_{2}}$, $q_{1}q_{2},\min\left\{q_{1}-2,q_{2}-2\right\},k_{1}k_{2})$	$k_{1}|q_{1}-1$, $k_{2}|q_{2}-1$, $\gcd(k_{1}\left(q_{1}-1\right),k_{2}\left(q_{2}-1\right))=1$, $k_{1}\left(q_{1}-1\right)<k_{2}\left(q_{2}-1\right)$ and $q_{1}>k_{1}k_{2}^{2}+2k_{1}k_{2}$, or $k_{1}\left(q_{1}-1\right)>k_{2}\left(q_{2}-1\right)$ and $q_{2}>k_{1}^{2}k_{2}+2k_{1}k_{2}$	[14]
$\left(\frac{q^{n}-1}{l},T,q^{k},Z_{4},\frac{q^{n-k}-1}{l}\right)$	n≥1, 0<k≤n, $q^{n}-1=T\left(Z_{4}+1\right)$, l>0, gcd(l,n)=1, l∣q−1	[18]
$\left(q^{n}-1,Tq^{k},q^{k},Z_{5},q^{n-k}\right)$	n≥1, 0<k≤n, $q^{n}-1=T\left(Z_{5}+1\right)$	[18]
$\left(\left(q_{3}-1\right)\left(q_{4}^{n}-1\right),q_{3},q_{3}q_{4},q_{4}^{n}-2,q_{4}^{n-1}-1\right)$	n≥1, $q_{3}>q_{4}^{n}$	[19]
$\left(T\iota_{1},k\eta_{1},\rho_{1},T-1,TH_{m}\left(G_{1}\right)\right)$	$uT=\iota_{1},k=u-1$, $\mathrm{RSLH}\left[T\iota_{1},k\eta_{1},\rho_{1},T-1\right]=T\mathrm{RSLH}\left[\iota_{1},\eta_{1},\rho_{1},\iota_{1}-1\right]$	[21]
$\left(q^{m}-1,nM\left(w-1\right),q^{k},l-n,q^{m-k}-1\right)$	$m\geq 1,0\leq k<m,l|\left(q-1\right),\gcd\left(l,m\right)=1,0<n\leq \left\lceil \frac{l}{2}\right\rceil$, $w>2l,M=\lceil \frac{q^{m}-1}{lw}\rceil$	[22]
(qL,qM,qv,L−1,1)	$q=p^{b},\gcd\left(q,L\right)=1,p>m,q>\geq 1$	[23]
$\left(\iota_{1}\lambda_{1},\eta_{1},\rho_{1},z_{1},\lambda_{1}H_{m}\left(G_{1}\right)\right)$	$\lambda_{1}\kappa_{1}=\iota_{1}$, $\gcd(\delta_{1},\iota_{1})=1$, $-\lambda_{1}<\delta_{1}<\lambda_{1}$, $z_{1}=\lambda_{1}\left(\kappa_{1}-1\right)+\delta_{1}-1$ if $0<\delta_{1}<\lambda_{1}$ or $z_{1}=\lambda_{1}\kappa_{1}+\delta_{1}-1$ if $-\lambda_{1}<\delta_{1}<0$, and $\mathrm{RSLH}\left[\iota_{1}\lambda_{1},\eta_{1},\rho_{1},z_{1}\right]=\lambda_{1}\mathrm{RSLH}\left[\iota_{1},\eta_{1},\rho_{1},\iota_{1}-1\right]$	Theorem 2
$\left(\iota_{1}\lambda_{2},\eta_{1},\rho_{1},\lambda_{2}\kappa_{2},\lambda_{2}H_{m}\left(G_{1}\right)\right)$	$2\leq \kappa_{2}\leq \left\lfloor \frac{\iota_{1}}{2}\right\rfloor$, $\kappa_{2}∤\iota_{1}$, $\lambda_{2}=\left\lfloor \frac{\iota_{1}}{\kappa_{2}}\right\rfloor$, $\mathrm{RSLH}\left[\iota_{1}\lambda_{2},\eta_{1},\rho_{1},\lambda_{2}\kappa_{2}\right]=\lambda_{2}\mathrm{RSLH}\left[\iota_{1},\eta_{1},\rho_{1},\iota_{1}-1\right]$	Theorem 3

*q*, $q_{1}$, $q_{2}$, $q_{3}$, $q_{4}$ are prime powers; *p*, $p_{1}$ are prime numbers; $G_{1}$ is an optimal FHS set with parameters $\left(\iota_{1},\eta_{1},\rho_{1},H_{m}\left(G_{1}\right)\right)$.

## Data Availability

Data are contained within the article.
